# Supplementary material for: End Sequence Analysis Toolkit (ESAT) expands the extractable information from single-cell RNA-seq data
Source: Genome Res. 2016 Oct;26(10):1397–410. doi: 10.1101/gr.207902.116 (PMC5052061; doi:10.1101/gr.207902.116)
Supplement: Supplemental Material [file supp_gr.207902.116_Supp_Materials.tar.gz › ESAT-esat_v0.1/doc/html/classumms_1_1core_1_1model_1_1score_1_1_basic_scan_statistic_score.html]

ESAT: umms.core.model.score.BasicScanStatisticScore Class Reference


ESAT


- Main Page
- Related Pages
- Packages
- Classes
- Files

- Class List
- Class Index
- Class Hierarchy
- Class Members

All Classes Namespaces Files Functions Variables Enumerator Pages

- umms
- core
- model
- score
- BasicScanStatisticScore

Classes |
Public Member Functions |
List of all members

umms.core.model.score.BasicScanStatisticScore Class Reference

Inheritance diagram for umms.core.model.score.BasicScanStatisticScore:

[legend]

Collaboration diagram for umms.core.model.score.BasicScanStatisticScore:

[legend]

|  |  |
| --- | --- |
| Classes | |
| class | **Processor** |
|  | |

|  |  |
| --- | --- |
| Public Member Functions | |
|  | BasicScanStatisticScore (AlignmentModel model, Annotation annotation, double score, boolean global, double regionLength, double regionScore) |
|  | |
|  | BasicScanStatisticScore (AlignmentModel model, Annotation annotation, double regionTotal, double regionLength2, boolean fullyContainedReads) |
|  | |
|  | BasicScanStatisticScore (AlignmentModel model, Annotation annotation, BasicScanStatisticScore previousScore, double newScore) |
|  | |
| double | calculateGlobalPvalue (AlignmentModel model) |
|  | |
| double | calculateLocalPvalue () |
|  | |
| double | getAverageCoverage () |
|  | |
| double | getWindowLength () |
|  | |
| void | setWindowLength (double length) |
|  | |
| double | getGlobalEnrichment () |
|  | |
| double | getLocalLambda () |
|  | |
| void | setGlobalLength (double d) |
|  | |
| double | getGlobalLambda () |
|  | |
| double | getGlobalLength () |
|  | |
| void | setRegionLength (double regionLength) |
|  | |
| double | getRegionLength () |
|  | |
| void | setScanPvalue (double scanPvalue) |
|  | |
| void | setPvalue (double scanPvalue) |
|  | |
| double | getScanPvalue () |
|  | |
| double | getPvalue () |
|  | |
| void | setFullyContainedNumberOfReads (double fullyContainedNumberOfReads) |
|  | |
| double | getFullyContainedNumberOfReads () |
|  | |
| double[] | getScores () |
|  | |
| String | toString () |
|  | |
| boolean | equals (Object o) |
|  | |
| Public Member Functions inherited from umms.core.model.score.CountScore | |
|  | CountScore (Annotation a) |
|  | |
|  | CountScore (CountScore other) |
|  | |
|  | CountScore (CountScore other, Annotation newAnnotation, double newCount) |
|  | |
|  | CountScore (AnnotationCollection<?extends Annotation > model, Annotation annotation) |
|  | |
|  | CountScore (AnnotationCollection<?extends Annotation > model, Annotation annotation, double regionTotal) |
|  | |
|  | CountScore (AnnotationCollection<?extends Annotation > model, Annotation annotation, boolean fullyContained) |
|  | |
|  | CountScore (AnnotationCollection<?extends Annotation > model, Annotation annotation, double regionTotal, boolean fullyContained) |
|  | |
|  | CountScore (Annotation annotation, double count, double regionTotal, double total) |
|  | |
| double | getCount () |
|  | |
| double | getScore () |
|  | |
| double | getTotal () |
|  | |
| double | getRegionTotal () |
|  | |
| double | getRPKM () |
|  | |
| double | getRPK () |
|  | |
| void | setCount (double count) |
|  | |
| void | setTotal (double total) |
|  | |
| void | setRegionTotal (double regionTotal) |
|  | |
| String | toString () |
|  | |
| boolean | equals (Object o) |
|  | |
| int | compareTo (CountScore o) |
|  | |
| int | hashCode () |
|  | |
| void | refreshPvalue () |
|  | |
| double | getPvalue () |
|  | |
| Public Member Functions inherited from umms.core.model.score.WindowScore.AbstractWindowScore | |
|  | AbstractWindowScore (Annotation t) |
|  | |
| Annotation | getAnnotation () |
|  | |

|  |  |
| --- | --- |
| Additional Inherited Members | |
| Static Public Member Functions inherited from umms.core.model.score.CountScore | |
| static double | asRPKM (double count, double total, int windowSize) |
|  | |
| Static Public Attributes inherited from umms.core.model.score.CountScore | |
| static double | DEFAULT\_REGION\_TOTAL = -1.0 |
|  | |
| Protected Attributes inherited from umms.core.model.score.WindowScore.AbstractWindowScore | |
| Annotation | annotation |
|  | |

## Constructor & Destructor Documentation

|  |  |  |  |
| --- | --- | --- | --- |
| umms.core.model.score.BasicScanStatisticScore.BasicScanStatisticScore | ( | AlignmentModel | *model*, |
|  |  | Annotation | *annotation*, |
|  |  | double | *score*, |
|  |  | boolean | *global*, |
|  |  | double | *regionLength*, |
|  |  | double | *regionScore* |
|  | ) |  |  |

Here is the call graph for this function:

Here is the caller graph for this function:

|  |  |  |  |
| --- | --- | --- | --- |
| umms.core.model.score.BasicScanStatisticScore.BasicScanStatisticScore | ( | AlignmentModel | *model*, |
|  |  | Annotation | *annotation*, |
|  |  | double | *regionTotal*, |
|  |  | double | *regionLength2*, |
|  |  | boolean | *fullyContainedReads* |
|  | ) |  |  |

BAD constructor

Parameters
:   |  |  |
    | --- | --- |
    | model |  |
    | annotation |  |
    | regionTotal |  |
    | regionLength2 |  |
    | fullyContainedReads |  |

|  |  |  |  |
| --- | --- | --- | --- |
| umms.core.model.score.BasicScanStatisticScore.BasicScanStatisticScore | ( | AlignmentModel | *model*, |
|  |  | Annotation | *annotation*, |
|  |  | BasicScanStatisticScore | *previousScore*, |
|  |  | double | *newScore* |
|  | ) |  |  |

BAD CONSTRUCTOR

Parameters
:   |  |  |
    | --- | --- |
    | model |  |
    | annotation |  |
    | previousScore |  |
    | newScore |  |

Here is the call graph for this function:

## Member Function Documentation

|  |  |  |  |  |  |
| --- | --- | --- | --- | --- | --- |
| double umms.core.model.score.BasicScanStatisticScore.calculateGlobalPvalue | ( | AlignmentModel | *model* | ) |  |

Here is the call graph for this function:

Here is the caller graph for this function:

|  |  |  |  |  |
| --- | --- | --- | --- | --- |
| double umms.core.model.score.BasicScanStatisticScore.calculateLocalPvalue | ( |  | ) |  |

Here is the call graph for this function:

Here is the caller graph for this function:

|  |  |  |  |  |  |
| --- | --- | --- | --- | --- | --- |
| boolean umms.core.model.score.BasicScanStatisticScore.equals | ( | Object | *o* | ) |  |

True iff scan P value and annotation are equal

Here is the call graph for this function:

|  |  |  |  |  |
| --- | --- | --- | --- | --- |
| double umms.core.model.score.BasicScanStatisticScore.getAverageCoverage | ( |  | ) |  |

Here is the call graph for this function:

Here is the caller graph for this function:

|  |  |  |  |  |
| --- | --- | --- | --- | --- |
| double umms.core.model.score.BasicScanStatisticScore.getFullyContainedNumberOfReads | ( |  | ) |  |

|  |  |  |  |  |
| --- | --- | --- | --- | --- |
| double umms.core.model.score.BasicScanStatisticScore.getGlobalEnrichment | ( |  | ) |  |

Here is the call graph for this function:

|  |  |  |  |  |
| --- | --- | --- | --- | --- |
| double umms.core.model.score.BasicScanStatisticScore.getGlobalLambda | ( |  | ) |  |

Here is the call graph for this function:

Here is the caller graph for this function:

|  |  |  |  |  |
| --- | --- | --- | --- | --- |
| double umms.core.model.score.BasicScanStatisticScore.getGlobalLength | ( |  | ) |  |

Here is the caller graph for this function:

|  |  |  |  |  |
| --- | --- | --- | --- | --- |
| double umms.core.model.score.BasicScanStatisticScore.getLocalLambda | ( |  | ) |  |

Here is the call graph for this function:

Here is the caller graph for this function:

|  |  |  |  |  |
| --- | --- | --- | --- | --- |
| double umms.core.model.score.BasicScanStatisticScore.getPvalue | ( |  | ) |  |

|  |  |  |  |  |
| --- | --- | --- | --- | --- |
| double umms.core.model.score.BasicScanStatisticScore.getRegionLength | ( |  | ) |  |

Here is the caller graph for this function:

|  |  |  |  |  |
| --- | --- | --- | --- | --- |
| double umms.core.model.score.BasicScanStatisticScore.getScanPvalue | ( |  | ) |  |

Here is the caller graph for this function:

|  |  |  |  |  |
| --- | --- | --- | --- | --- |
| double [] umms.core.model.score.BasicScanStatisticScore.getScores | ( |  | ) |  |

Here is the call graph for this function:

|  |  |  |  |  |
| --- | --- | --- | --- | --- |
| double umms.core.model.score.BasicScanStatisticScore.getWindowLength | ( |  | ) |  |

Here is the caller graph for this function:

|  |  |  |  |  |  |
| --- | --- | --- | --- | --- | --- |
| void umms.core.model.score.BasicScanStatisticScore.setFullyContainedNumberOfReads | ( | double | *fullyContainedNumberOfReads* | ) |  |

|  |  |  |  |  |  |
| --- | --- | --- | --- | --- | --- |
| void umms.core.model.score.BasicScanStatisticScore.setGlobalLength | ( | double | *d* | ) |  |

Here is the caller graph for this function:

|  |  |  |  |  |  |
| --- | --- | --- | --- | --- | --- |
| void umms.core.model.score.BasicScanStatisticScore.setPvalue | ( | double | *scanPvalue* | ) |  |

|  |  |  |  |  |  |
| --- | --- | --- | --- | --- | --- |
| void umms.core.model.score.BasicScanStatisticScore.setRegionLength | ( | double | *regionLength* | ) |  |

Here is the caller graph for this function:

|  |  |  |  |  |  |
| --- | --- | --- | --- | --- | --- |
| void umms.core.model.score.BasicScanStatisticScore.setScanPvalue | ( | double | *scanPvalue* | ) |  |

Here is the caller graph for this function:

|  |  |  |  |  |  |
| --- | --- | --- | --- | --- | --- |
| void umms.core.model.score.BasicScanStatisticScore.setWindowLength | ( | double | *length* | ) |  |

Here is the caller graph for this function:

|  |  |  |  |  |
| --- | --- | --- | --- | --- |
| String umms.core.model.score.BasicScanStatisticScore.toString | ( |  | ) |  |

Here is the call graph for this function:

---

The documentation for this class was generated from the following file:

- java/umms/core/model/score/BasicScanStatisticScore.java


---

Generated on Fri Aug 15 2014 15:11:12 for ESAT by  

 1.8.7
